# Repurposing the phospholipase A2 inhibitor, darapladib, to combat multidrug-resistant fungal pathogens, including *Candida auris*

**DOI:** 10.1371/journal.pone.0361366

**Published:** 2026-10-26

**Authors:** Yehia Elgammal, Ehab A. Salama, Nour M. Alkashef, Christopher B. Lawrence, Mohamed N. Seleem

**Affiliations:** 1 Department of Biomedical Sciences and Pathobiology, Virginia-Maryland College of Veterinary Medicine, Virginia Polytechnic Institute and State University, Blacksburg, Virginia, United States of America; 2 Center for One Health Research, Virginia Polytechnic Institute and State University, Blacksburg, Virginia, United States of America; Kwame Nkrumah University of Science and Technology, GHANA

## Abstract

*Candida auris* is an emerging multidrug-resistant fungal pathogen and a major global health threat, highlighting the urgent need for new therapeutics. We screened ~2,600 FDA-approved drugs and clinical compounds and identified darapladib (DAL), a phospholipase A2 inhibitor, as a potent antifungal agent against multidrug-resistant *C. auris*. DAL exhibited antifungal activity against all tested *C. auris* isolates and showed broad-spectrum activity against other tested clinically important fungal pathogens, including other *Candida*, *Cryptococcus*, and mucormycosis-associated species, with minimum inhibitory concentrations of 4–16 μg/mL. DAL also displayed rapid fungicidal activity against *C. auris* and synergized with amphotericin B against *C. auris* and filamentous fungal pathogens. More importantly, serial exposure of *C. auris* to subinhibitory concentrations of DAL for 16 passages did not result in detectable resistance. Mechanistic studies indicated that DAL acts through an ergosterol-dependent membrane targeting mechanism, as exogenous ergosterol attenuated its activity and ergosterol-related haploinsufficient mutants showed reduced susceptibility. Consistent with this mechanism, DAL increased membrane permeability, nucleic acid leakage, and reactive oxygen species. In a *Caenorhabditis elegans–C. auris* infection model, DAL reduced the *C. auris* burden to below the limit of detection. Together, these findings support further investigation of DAL as a repurposed antifungal candidate against drug-resistant fungal infections.

## Introduction

Invasive candidiasis, an infection caused by *Candida* species, causes significant hospital-acquired morbidity and mortality worldwide [1,2]. It represents a major source of healthcare-associated sepsis, especially in intensive care units, where it poses life-threatening risks in individuals with compromised immune systems [3]. The emergence of multidrug-resistant *Candida* species, particularly *Candida auris,* has been recognized as a global health threat due to its ability to cause invasive infections with alarming mortality rates (30–60%) [4]. In the United States, *Candida* species cause up to 22% of healthcare-associated bloodstream infections with approximately 25,000 cases of candidemia each year [2,5,6]. While *C. albicans* remains the most common pathogenic species, other non-*albicans* species are also prevalent [2]. Since its emergence in 2009, *C. auris* has become a significant global concern due to its ability to develop multidrug-resistance and rapid transmission within healthcare settings [7]. The rising prevalence of *C. auris* across healthcare facilities worldwide underscores the urgent need for novel antifungal therapies [8,9]. Without advancements in treatment, *C. auris* is likely to continue its rapid global spread, increasing morbidity and mortality rates among vulnerable patient populations and healthcare systems [10].

*C. auris* resistance to major classes of antifungals, including azoles, polyenes and echinocandins, presents a significant treatment challenge and highlights issues in clinical management [11]. In *C. auris* clinical isolates, fluconazole resistance is especially high, exceeding 90%, while resistance to polyenes reaches over 30%. In contrast, resistance to echinocandins remains relatively low at 5%, making echinocandins the preferred first-line therapy [12–14]. Nevertheless, several reports have already identified pan-resistant *C. auris* isolates, underscoring the need for novel therapeutic options as multidrug resistance continues to emerge in the clinical environment [15].

Pathogenic fungi responsible for invasive aspergillosis and mucormycosis frequently exhibit resistance to current antifungals, presenting a significant clinical challenge due to high mortality rates [16]. Treatment options are further limited by the toxicity of available antifungal drugs, complicating patient management. *Aspergillus* species, particularly *A. fumigatus*, are primary pathogens responsible for invasive aspergillosis, a life-threatening disease primarily affecting severely immunocompromised individuals, such as cancer patients and organ transplant recipients [17,18]. Despite advancements in antifungal therapies and improved treatment approaches, aspergillosis continues to have a high mortality rate, remaining around 35% [19]. Additionally, the rising prevalence and global spread of azole-resistant *A. fumigatus* strains present an ongoing challenge to healthcare worldwide. Mucormycosis is another highly aggressive fungal infection with devastating outcomes. Global incidence is estimated at around 910,000 cases annually, with mortality rates often exceeding 40–50%, and reaching >80% in disseminated disease or delayed treatment [20,21]. It is primarily caused by *Rhizopus* and *Mucor* species from the *Mucorales* order [22]. Treatment generally involves surgical intervention, management of underlying health conditions, and systemic antifungal therapy, with liposomal amphotericin B (AmB) as the most effective option [23,24]. However, many *Mucorales* species and other closely-related species causing mucormycosis show resistance to standard antifungal treatments, including AmB, which often have significant side effects. These challenges highlight the urgent need for safer and more effective therapeutic options.

To address this unmet need, we conducted a high-throughput screening of ~2,600 FDA-approved drugs and clinical compounds and identified darapladib (DAL) as a potent inhibitor of *C. auris* growth. DAL is an oral potent inhibitor of human lipoprotein-associated phospholipase A2 (Lp-PLA2) [25–27]. This enzyme, which is often released into the bloodstream by monocytes/macrophages, possesses pro-inflammatory and oxidative properties. It contributes to the accumulation of low-density lipoprotein (LDL) cholesterol, promoting the formation of atherosclerotic plaques and increased risk of cardiovascular events. Originally developed for the treatment of atherosclerosis, DAL was well tolerated in Phase I and II clinical trials with no significant safety concerns but it was discontinued during Phase III trials due to lack of efficacy in reducing major cardiovascular events [28,29]. While DAL successfully inhibited Lp-PLA2, clinical trials showed that this inhibition did not result in significant improvements in reducing cardiovascular events when compared to standard treatments [28,30]. Although DAL was discontinued for atherosclerosis treatment, its well-established safety and pharmacokinetic profiles have generated interest in repurposing it for other therapeutic applications, including Diabetic Macular Edema (DME), Alzheimer’s Disease, and various inflammatory conditions [31–33]. DAL has previously reported to exhibit antifungal activity against *Candida* biofilm [34]. Although DAL is a known phospholipase A2 inhibitor, there was also no direct evidence linking its antifungal activity to fungal phospholipase inhibition.

In this study, we evaluated the phospholipase A2 inhibitor (PLA2), DAL as a repurposed antifungal candidate against multidrug-resistant *C. auris* and other clinically important fungal pathogens. We assessed its fungicidal activity, interaction with AmB, susceptibility after serial exposure, impact on fungal membrane integrity and reactive oxygen species (ROS), and *in vivo* efficacy in a *Caenorhabditis elegans* infection model. We further investigated the potential involvement of ergosterol in DAL activity using exogenous ergosterol supplementation and ergosterol-related heterozygous deletion (haploinsufficiency) *C. albicans* mutants. We hypothesized that DAL exerts its antifungal activity through ergosterol associated disruption of fungal membrane and potentiates the antifungal activity of AmB.

## Materials and methods

### Fungal isolates, media, chemicals, and compounds

Nineteen fungal isolates (10 *C. auris*, 1 *C. albicans*, 1 *C. krusei,* 1 *C. parapsilosis*, 1 *C. tropicalis*, 2 *C. neoformans*, 1 *C. gatti, 1 R. arrhizus, and 1 M. indicus*) were acquired from the CDC (Atlanta, GA, USA), BEI Resources (Manassas, VA, USA), and Westerdijk Fungal Biodiversity Institute (Utrecht, Netherlands) and used in our study. Supplementary S1 and S2 Tables provide the sources of all fungal isolates and clade classifications of *C. auris* isolates.

Media, reagents, and compounds were purchased from the following suppliers: RPMI 1640 and Dulbecco’s Modified Eagle Medium (DMEM, Gibco, Grand, Island, NY, USA); 3-(N-Morpholino) propane sulfonic acid (MOPS, Fisher Bioreagents, Fairlawn, NJ, USA); yeast extract peptone dextrose agar and broth (YPD, Becton, Dickinson, and Company, Sparks, MD, USA); phosphate-buffered saline (PBS) and fetal bovine serum (FBS, Corning, Manassas, VA, USA); AmB (Chem-Impex International, Wood Dale, IL, USA); 2′,7′-dichlorofluorescin diacetate (DCFH-DA), ergosterol and phenazine methosulfate (PMS, Thermo Fisher Scientific Inc, Waltham, MA, USA); MTS (3-(4,5-dimethylthiazol-2-yl)-5-(3-carboxymethoxyphenyl)-2-(4-sulfophenyl)-2H-tetrazolium, Promega, Madison, WI, USA); DAL (MuseChem, Paoli, PA, USA); gentamicin sulfate and propidium iodide (PI; Sigma-Aldrich, St. Louis, MO, USA).

### High-Throughput screening

The Drug Repurposing Compound Library (HY-L035, MedChemExpress), which contains 2,600 FDA-approved drugs and clinical compounds, was screened at a final concentration of 16 µM against *C. auris* AR0390 in RPMI 1640 medium buffered with MOPS medium, using a fungal inoculum standardized to approximately 1 × 10^3^ CFU/mL. After incubation at 35 °C for 24 hours, optical density was measured at 490 nm with a BioTek Synergy H1 microplate reader. Growth was expressed as a percentage relative to the untreated control, and compounds causing ≥80% growth inhibition were considered potential hits. Untreated *C. auris* cells served as the negative growth control, and AmB and fluconazole were included as positive controls. The library screening was performed as a single screening, and identified hits were subsequently validated by MIC testing in duplicate across three independent experiments. Hits were evaluated individually based on confirmed antifungal activity and available pharmacokinetic properties, and DAL was prioritized for further characterization based on this assessment.

### MIC and checkerboard assays

The minimum inhibitory concentrations (MICs) of DAL were determined using the CLSI standard broth microdilution methods for yeasts and filamentous fungi [35,36]. Briefly, susceptibility testing was performed in RPMI-MOPS. For yeasts (*Candida* and *Cryptococcus*), the final inoculum was standardized to 1 × 10^3^ colony-forming units (CFU)/mL, whereas for filamentous fungi (*Aspergillus* and mucormycosis-associated species), the final inoculum was adjusted to 2 × 10^4^ CFU/mL. Interactions between DAL and AmB were evaluated against *Candida, Aspergillus, and* Mucormycosis-associated species using checkerboard assay [36]. The fractional inhibitory concentration index (FICI) was calculated using MIC_100_ endpoints and used to interpret interactions indicating synergistic (FICI ≤ 0.5), indifferent (0.5 < FICI < 2), or antagonistic effects (FICI > 2) [37].

### Time-kill assay

To evaluate the killing kinetics of DAL, an inoculum of 10⁴ CFU/mL of *C. auris* strains AR0390 and AR0389 was prepared in RPMI broth [38,39]. *C. auris* cells were suspended in tubes and treated with DAL at 2 × MIC. At specified time points (0, 2, 4, 6, 12, 24, 30, 36, and 48 hours) during incubation at 35°C, 100 μl aliquots were vortexed and transferred into 96-well plate. The cells were then serially diluted, plated onto YPD agar, and incubated for 24 hours. CFUs were then counted to quantify viable cells. To evaluate the fungicidal activity of the combination, a time-kill assay was performed using DAL and AmB at sub-inhibitory concentrations. Additionally, the growth kinetics of *R. arrhizus* and *M. indicus* were further evaluated at several time points in the presence of AmB (1 μg/mL), DAL (4 μg/mL), or the combination of both drugs. The optical density (OD_600_) was measured at defined time points to assess the fungal growth over time, compared to the untreated control.

### Cytotoxicity of the combination AmB/DAL

Cytotoxicity of the combination AmB/DAL was assessed using the Vero (kidney epithelial cells extracted from an African green monkey) cell line [40,41]. Cells were cultured in DMEM supplemented with 10% FBS and incubated at 37°C with 5% CO_2_ for 24 hours to allow monolayer formation. Adherent cells were then treated with DAL (4 µg/mL), AmB (1 µg/mL), or a combination of DAL/AmB and incubated at 37°C for an additional 24 hours. Cell viability was determined by adding a mixture of MTS/PMS for 3 hours, followed by measuring the absorbance at 490 nm.

### Multistep resistance

We have investigated the development of resistance to DAL in *C. auris* by repeatedly exposing the organism to sub-inhibitory concentrations of the drug [42]. Briefly, *C. auris* AR0390 was grown overnight in 15 ml of YPD broth. An inoculum 1  ×  10^3^ of *C. auris* was prepared in duplicate, with duplicate cultures maintained as independent experiments. Cultures were exposed to sub-inhibitory concentrations of DAL, and 5-FC, as a positive control, at 0.25 × MIC into RPMI. Untreated cultures were included as negative control. Both untreated and drug-treated cultures were incubated at 35°C for 24 hours, with MIC values reassessed every two passages. This process was conducted over a total of 16 passages to monitor any shifts in susceptibility, providing insight into the potential development of drug resistance over repeated exposures.

### Membrane permeabilization using propidium iodide

Fungal membrane permeability was assessed by measuring the fluorescence intensity of PI [43,44]. Briefly, *C. auris* were grown overnight in YPD and diluted to a concentration of 1 × 10^7^ CFU/mL in RPMI. The fungal suspensions were treated with DAL at dose-dependent concentrations (at 2 × , 4 × , and 8 × MIC) and incubated at 37°C for 3 hours. After incubation, the cells were washed by centrifugation, re-suspended in sterile PBS, and treated with PI (10 μM). The mixture was vortexed and incubated 37°C for 30 minutes. Following incubation, the cells were washed again by centrifugation and resuspended in PBS. Fluorescence intensity was measured using a Biotek Synergy H1 microplate reader with excitation/emission wavelengths of 535nm/615nm to assess the plasma membrane damage. Statistical comparisons between groups were conducted using One-way ANOVA, followed by Dunnett-t test in GraphPad.

### Nucleic acid leakage assay

The effect of DAL on nucleic acid leakage from *C. auris* cells was assessed using a NanoDrop spectrophotometer, as previously reported with minor modifications [45]. Briefly, *C. auris* cells (1 × 10^7^ CFU/mL) were prepared from an overnight culture and exposed to dose-dependent concentrations of DAL for 3 hours. After treatment, the supernatant from each treatment was collected and analyzed to quantify nucleic acid content, comparing it to the nucleic acid levels in the supernatant from untreated control samples.

### ROS assay

ROS levels were measured using a fluorometric assay with DCFH-DA, following a slightly modified protocol [44,46]. Briefly, *C. auris* cells (1 × 10^7^ cells/mL) were treated with 2 × MIC of DAL, and AmB (as a positive control) for 3 hours at 35°C with constant shaking. After incubation, DCFH-DA dye (10 μM) was added to each cell suspension to detect ROS production. Fluorescence intensity, reflecting intracellular ROS levels, was measured using a microplate reader with excitation and emission wavelengths set at 485 nm and 530 nm, respectively. Untreated cells served as the negative control. Results are based on two independent experiments, with data reported as means.

### Effect of exogenous ergosterol and cholesterol on the MICs and killing kinetics of DAL

The MICs of DAL and AmB (positive control) were determined by the broth microdilution method, and their killing kinetics were evaluated in parallel against *C. auris* AR390 in the presence and absence of exogenous ergosterol or cholesterol (50, 100 and 200 μg/mL) [43].

### Heterozygous mutant analysis

A panel of 31 *C. albicans* ergosterol related deficient mutants was adjusted to 1 × 10^3^ CFU/mL in RPMI, treated with DAL at 2 × MIC (16 μg/mL) and incubated at 35 °C for 24 h. The parent strain (*C. albicans* SC5314) was used as a control. After incubation, treated strains were serially diluted in PBS and plated onto YPD agar. Growth of each mutant strain was compared with that of the parental strain to evaluate relative susceptibility to DAL. Reduced susceptibility was defined with greater than 2 log_10_ difference relative to the parental strain following DAL exposure.

### *C. auris-C. elegans* infection model

To assess the efficacy of DAL *in vivo*, we employed the *C. elegans* infection model as previously described by our group [47]. In brief, *C. elegans* AU37 (*Δglp-4; Δsek-1*) worms were grown, seeded in nematode growth media (NGM) agar plates with *E. coli* strain OP50 and synchronized to the larval stage day 4 (L4) using the alkaline bleach method [48]. The synchronized worms were infected with *C. auris* AR0390 (1 × 10^7^ CFU/mL) for 4 hours. Following the infection period, the worms were washed four times with PBS, divided into treatment groups in M9 buffer supplemented with 20% RPMI, and treated with 2 × MIC of DAL, and itraconazole (ITC). An untreated group was included as a negative control. After 24 hours of treatment at room temperature, worms were examined microscopically to confirm viability, then they were collected, washed with PBS, and homogenized using silicon carbide beads for 15 minutes. The resulting homogenates were serially diluted, plated on YPD agar plates containing gentamicin (100 µg/mL), and CFUs were counted after 24 hours of incubation at 35°C.

### Statistical analysis

Statistical analyses were performed using GraphPad Prism. Data are presented as mean ± SD, and *P* < 0.05 was considered statistically significant.

### Ethics Statement

The authors have nothing to report.

## Results

### Identification of DAL as a novel antifungal agent

We screened a Drug Repurposing Compound Library (HY-L035, MedChemExpress), which contains ~2,600 approved drugs and clinical compounds, against *C. auris* AR0390 (South Asia, clade I isolate), to identify potential drugs that could be repurposed as antifungals. Compounds that inhibited ≥80% of *C. auris* growth at 16 μM in the screening were considered primary hits. The identified hits were subsequently evaluated based on its confirmed antifungal activity and available pharmacokinetic properties. Based on these criteria, DAL was prioritized for further characterization (Fig 1). Therefore, we investigated the *in vitro* and *in vivo* antifungal activity of DAL, highlighting the potential of PLA2 inhibitors as effective therapeutic options against *C. auris*.

**Fig 1:**
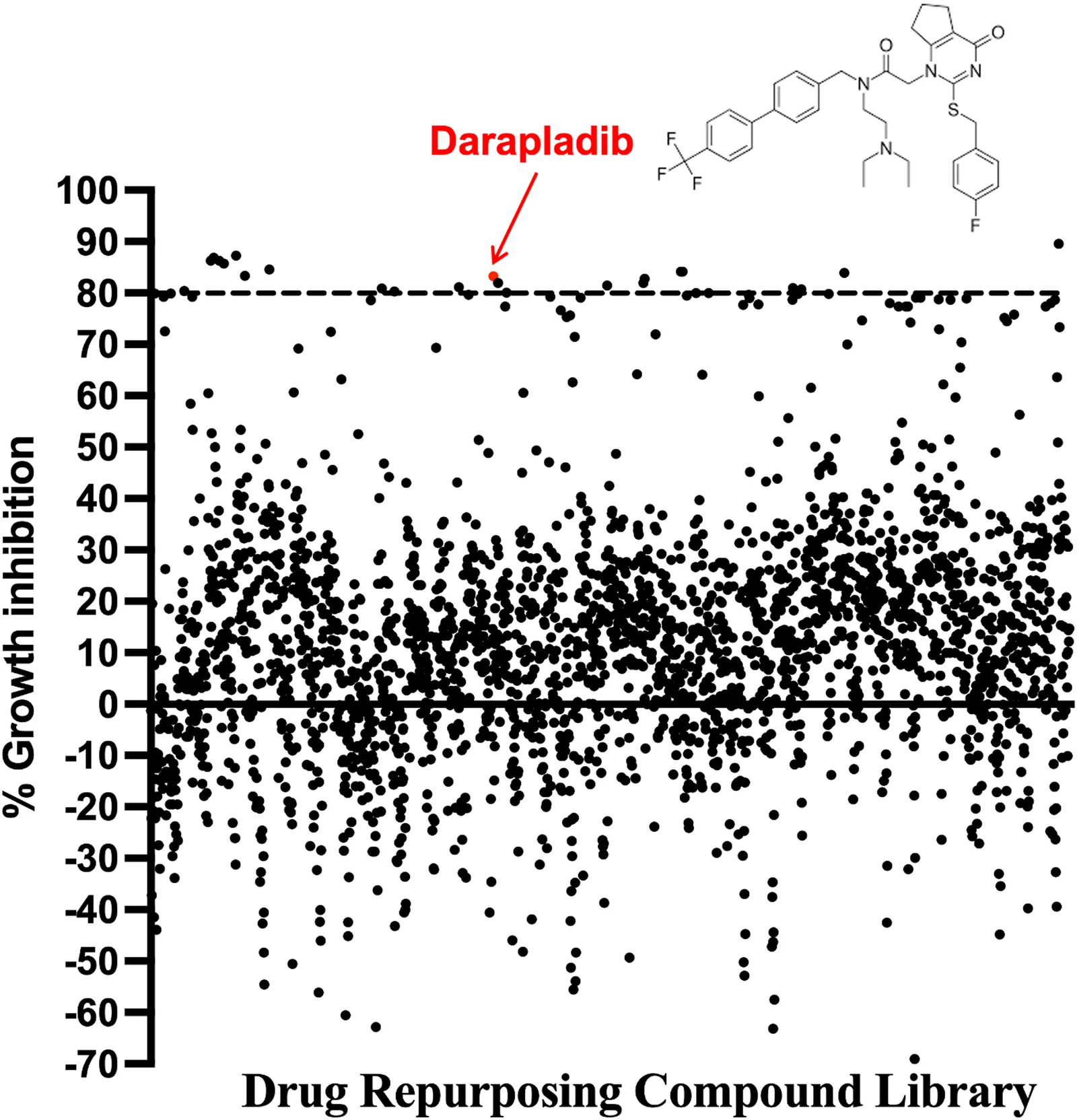
Drug-repurposing library screening against *C. auris.* Screening of the drug-repurposing compound library (~2,600 compounds; HY-L035, MedChemExpress) at 16 µM against *C. auris* AR0390. Compounds producing ≥80% growth inhibition relative to the untreated growth control were considered primary hits. Darapladib was selected as potent inhibitor for further investigation.

We tested the activity of DAL against several isolates of *C. auris* (*n* = 10) where it exhibited antifungal activity against all tested *C. auris* isolates (MICs = 8 μg/mL) (Table 1). Additionally, DAL inhibited the growth of other fungal pathogens, including *Candida*, *Cryptococcus*, and mucormycosis-associated species with MICs ranging from 4–16 μg/mL. In a standard time-kill assay, DAL exhibited a rapid fungicidal effect, eradicating *C. auris* cells within 2 hours (Fig 2A and B). Moreover, DAL at sub-inhibitory concentrations potentiated the antifungal activity of AmB and cleared the CFU of *C. auris* within 6 hours (Fig 2C and D).

**Table 1:** Minimum inhibitory concentrations (MICs) of darapladib (DAL), amphotericin B (AmB), and fluconazole (FLC) against fungal isolates.

Fungal isolate	MIC (µg/mL)		
	DAL	AmB	FLC
***C. auris* AR0381**	8	1	1
***C. auris* AR0382**	8	1	1
***C. auris* AR0383**	8	1	128
***C. auris* AR0384**	8	1	>128
***C. auris* AR0385**	8	2	>128
***C. auris* AR0386**	8	2	>128
***C. auris* AR0387**	8	1	1
***C. auris* AR0388**	8	2	>128
***C. auris* AR0389**	8	2	>128
***C. auris* AR0390**	8	2	>128
***C. albicans* ATCC® 64124**	8	1	>128
***C. krusei* ATCC®34135**	8	2	16
***C. parapsilosis* ATCC® 22019**	4	0.5	2
***C. tropicalis* ATCC® 1369**	8	1	1
***C. neoformans* NR-41298**	4	1	2
***C. neoformans* NR-41300**	8	1	2
***C. gatti* NR-43208**	8	1	4
***Rhizopus arrhizus* var. delemar CBS120593**	16	2	>128
***Mucor indicus* CBS123974**	16	1	>128

**Fig 2:**
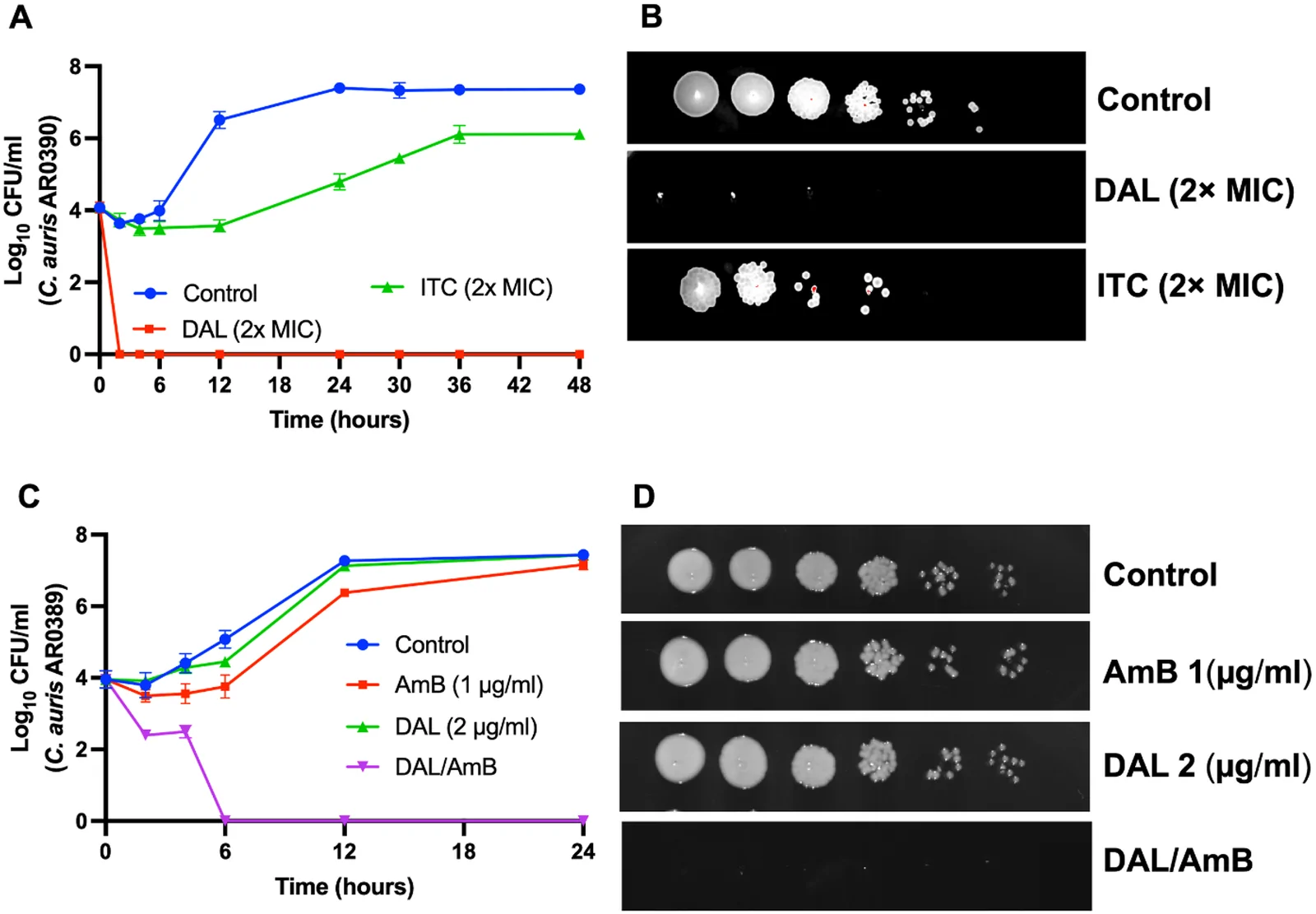
Killing kinetics of darapladib against *C. auris.* (A, B) Time-kill assay of darapladib (DAL,16 µg/mL), and itraconazole (ITC, 2 µg/mL), which is equivalent to 2 × MIC against *C. auris* AR0390. (C, D) Time-kill assays of *C. auris* AR0389 treated with sub-inhibitory concentrations of DAL (2 µg/mL), AmB (1 µg/mL), or the combination of both drugs. Fungal load (Log_10_ CFU/mL) was counted over time. For A-C, each symbol represents an average of 3 replicates per time point. These experiments were performed twice, and similar results were obtained.

### DAL interacts synergistically with AmB against several fungal pathogens

We examined the synergistic interaction of DAL with AmB against several fungal pathogens, including *C. auris, Aspergillus* and mucormycosis-associated species (*Rhizopus, Mucor,* and *Lichtheimia* spp.) and observed synergistic activity with ΣFICI ranges from 0.25 to 0.5 (Fig 3A, and B). The growth kinetics of *R. arrhizus* and *M. indicus* isolates further supported these findings. The combination of AmB and DAL (1 μg/mL and 4 μg/mL, respectively) resulted in a significant reduction of the fungal growth over time, showing improved antifungal activity compared to each drug alone (Fig 3C). This synergistic effect of DAL and AmB across multiple fungal species supports a potential strategic approach whereby one could decrease the required AmB dose, reduce associated toxicity, and improve overall treatment efficacy in a clinical setting.

**Fig 3:**
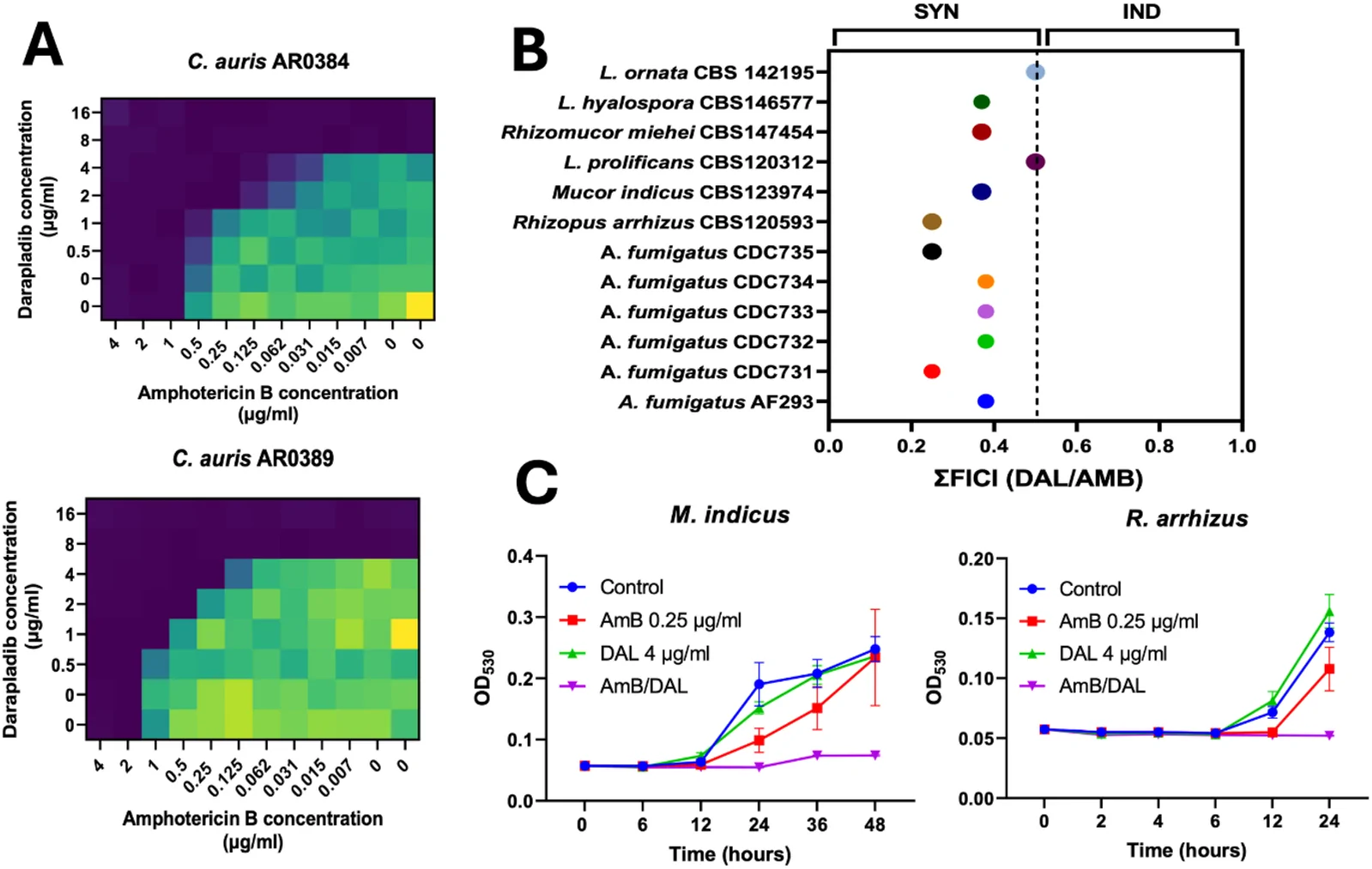
Interaction of darapladib (DAL) with amphotericin B (AmB) against different fungal pathogens. A) Heat maps of the synergistic interactions between DAL and AmB against AmB-susceptible (AR0384) and AmB-resistant (AR0389) *C. auris* isolates. B) ΣFICI of the combination AmB/DAL against filamentous fungal pathogens. C) Growth curves of *Mucor indicus* CBS123974 and *Rhizopus arrhizus* CBS120593 in the presence of sub-inhibitory concentrations of AmB (0.25 µg/mL), DAL (4 µg/mL) or their combination (*n*  =  3). These experiments were performed twice, and similar results were obtained.

### DAL susceptibility remains stable after serial exposure

After confirming the antifungal activity of DAL, we investigated the potential for *C. auris* resistance development. A multi-step resistance selection experiment was conducted with DAL, and 5-fluorocytosine (5-FC, as a positive control) against *C. auris* AR0390 (Fig 4A). After 16 passages, no increase in the MICs of DAL was observed, suggesting that *C. auris* is unlikely to develop rapid resistance to DAL. In contrast, the MIC for 5-FC increased more than 500-fold compared to the initial MIC value.

**Fig 4:**
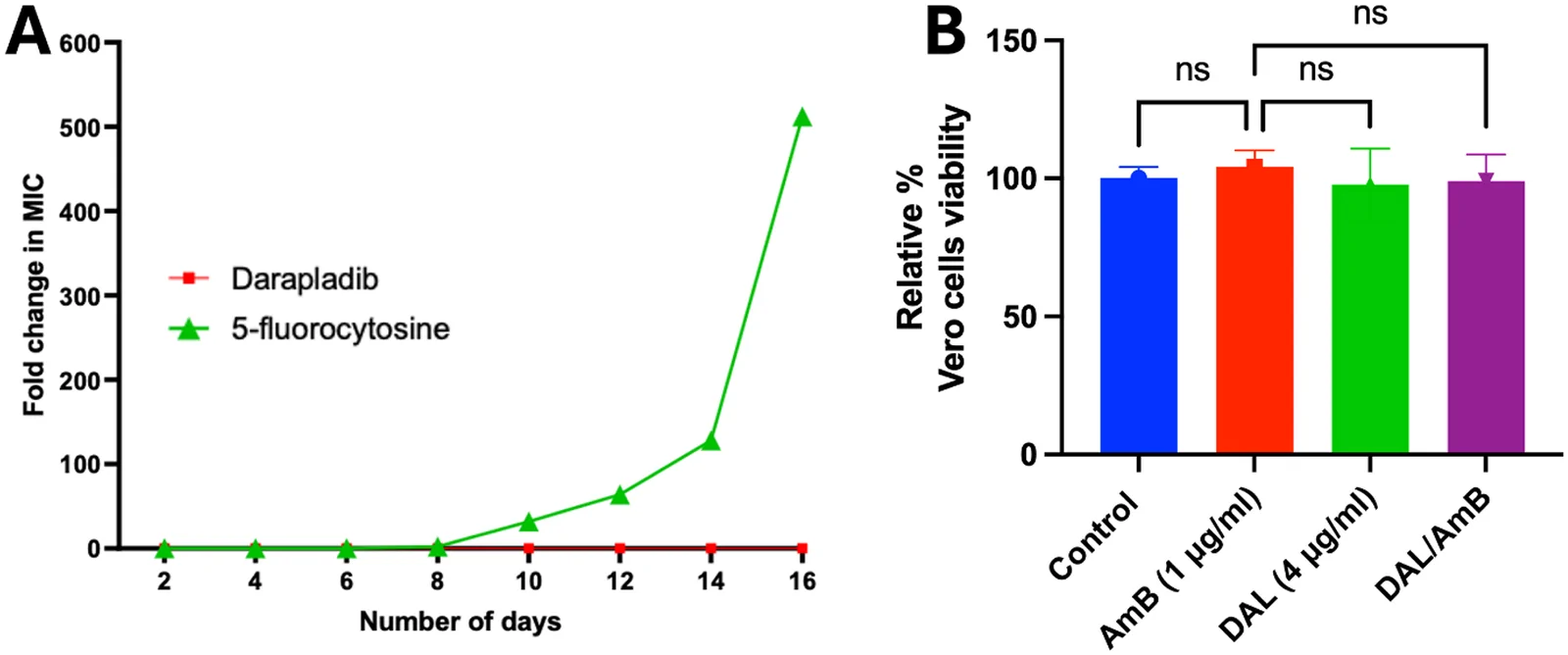
Resistance development and *in vitro* cytotoxicity of darapladib (DAL). A) Multistep resistance of DAL, and 5-fluorocytosine (5-FC) against *C. auris* AR0390 over 16 passages*. C. auris* cultures AR390 were exposed to sub-inhibitory concentrations of each drug (0.25 × MIC), and MICs were determined every two passages. Fold changes relative to the initial MIC were calculated to assess resistance development over successive generations. B) *In vitro* cytotoxicity of DAL alone and in combination of amphotericin B (AmB) against Vero (kidney) cells. Cells were treated with AmB (1 µg/mL), DAL (4 µg/mL), or a combination of both, and the cell viability was assessed using the Non-Radioactive Cell Proliferation (MTS) assay. DMSO was used as a negative control, and data represent the percent cell viability relative to the negative control, with error bars indicating standard deviation values. These experiments were performed twice, and similar results were obtained. No statistically significant difference was observed among the groups (ns = not significant).

### DAL does not increase the cytotoxic effects of AmB on kidney (Vero) cells

Cell viability assays showed no significant difference in viability between cells treated with AmB alone and those exposed to the combination of AmB (1 μg/mL) and DAL (4 μg/mL) (Fig 4B). These results suggest that the observed synergy in the antifungal activity between AmB and DAL is unlikely to result in elevated toxicity towards cells *in vitro*.

### The interaction between DAL and ergosterol in *C. auris* suggests an ergosterol-dependent fungicidal mechanism of action

To investigate the impact of ergosterol in the antifungal activity of DAL, we performed an ergosterol supplementation assay against *C. auris* [49–51]. Interestingly, the addition of exogenous ergosterol (50–200 µg/mL) in a broth microdilution assay reduced the antifungal activity of DAL, as indicated by a 4-fold increase in MIC values, similar to the effect observed with AmB (Fig 5A). Furthermore, a time-kill assay demonstrated that *C. auris* cells treated with DAL in the presence of ergosterol (200 µg/mL) partially restored the growth and exhibited fungistatic activity over 24 hours, compared to the cells treated in the absence of ergosterol (Fig 5B). To further assess whether DAL activity is linked to ergosterol, a panel of *C. albicans* heterozygous deletion (haploinsufficiency) mutants related to ergosterol biosynthesis was evaluated. These mutants are part of the Merck *C. albicans* double barcoded collection (DBC). It is most commonly used in the genome-wide fitness test as first reported by Xu et al. [52], and has been shown to be very effective in identifying the mode of action of various antifungal compounds [53–55]. The panel included 31 heterozygous deletion mutants corresponding to genes involved in the ergosterol biosynthesis. Among these, 9 mutants (*ERG26*, *ERG10*, *NCP1*, *ERG1*, *ERG27*, *ERG3*, *ARE2, ERG12*, and *IDI1*) showed lower susceptibility when treated with DAL at 2 × MIC (Fig 5C). Collectively, these findings further supported the association between DAL activity and ergosterol.

**Fig 5:**
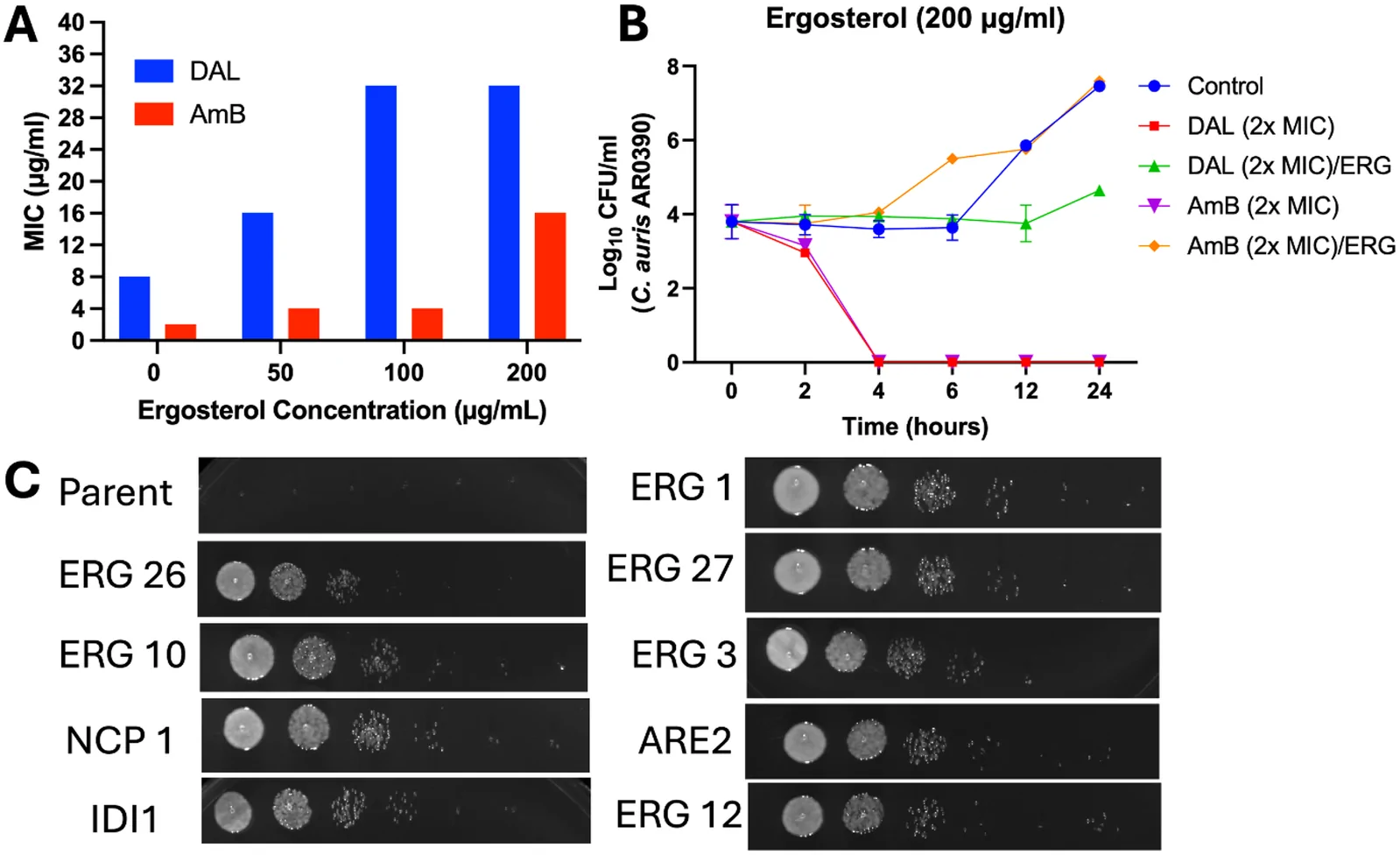
DAL activity is associated with ergosterol in *C. auris.* Effect of exogenous ergosterol (50-200 µg/mL) on the MICs (A) and time-kill (*n*  =  3) (B) of darapladib (DAL) and amphotericin B (AmB) at 2 × MIC against *C. auris* AR0390. C) Susceptibility of *C. albicans* heterozygous deletion mutants with disruptions in genes involved in ergosterol biosynthesis pathways following exposure to DAL (2 × MIC) for 24 h. Mutants treated with DAL were serially dilution and plated on YPD agar. The growth was assessed relative to the parent strain.

### DAL causes membrane permeabilization and nucleic acid leakage, and increases ROS levels

The association between DAL and ergosterol, as suggested by competition assays, is expected to increase plasma membrane permeability in *C. auris,* leading to nucleic acid leakage, ROS production, and subsequent cell death. To confirm this, we performed plasma membrane permeability and nucleic acid leakage assays and measured the ROS levels of *C. auris* exposed to DAL. All results were normalized to the untreated control, which was defined as 100%. Cells exposed to DAL exhibited dose-dependent increases in plasma membrane permeability (527%, 1,096%, and 1,875% at 2 × , 4 × , and 8 × MIC, respectively), accompanied by DNA leakage (141%, 225%, and 284%, respectively) and elevated ROS levels (490%) at 2 × MIC, relative to the untreated control cells (Fig 6A-C).

**Fig 6:**
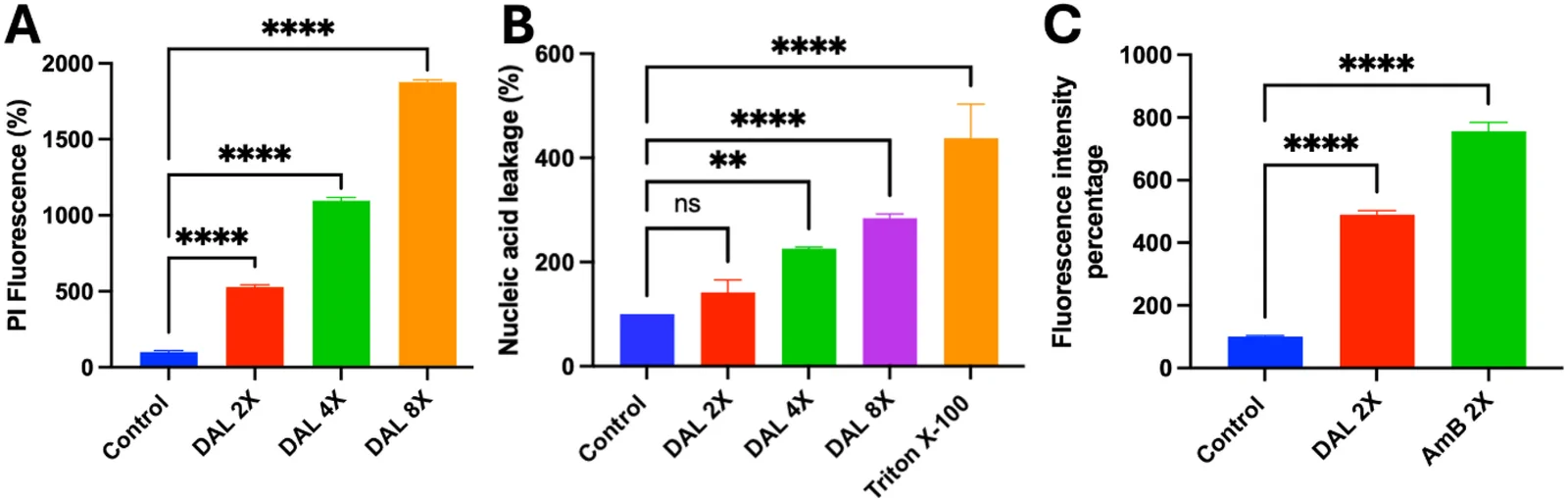
Evaluation of plasma membrane integrity, nucleic acid leakage, and ROS generation in *C. auris* following treatment with darapladib (DAL). (A) Assessment of *C. auris* AR0390 plasma membrane integrity when treated with DAL using a propidium iodide (PI) influx assay to measure cell permeability changes. (B) Quantification of nucleic acid leakage from cells treated with DAL using a NanoDrop spectrophotometer to detect extracellular nucleic acid levels. (C) ROS generation induced by DAL, and amphotericin B (AmB, as positive control) treatment in *C. auris*. These experiments were performed twice, and similar results were obtained. Statistical significance was determined using a One-way ANOVA. * indicates significant differences between treatments compared to control (**P < 0.01, **** *P* < 0.0001).

### DAL retains fungicidal activity in the presence of exogenous cholesterol

Due to concerns about toxicity, particularly because AmB binds to cholesterol in human renal cells, we performed a cholesterol supplementation assay [56–58]. The MIC of DAL increased only by 2-fold (Fig 7A), compared to the 4-fold increase in MIC of AmB in the presence of cholesterol. Moreover, DAL retained its fungicidal activity even in the presence of cholesterol, whereas AmB lost its activity (Fig 7B). These findings indicate that DAL activity is less affected by exogenous cholesterol than AmB and is more strongly associated with ergosterol than with cholesterol under the conditions tested.

**Fig 7:**
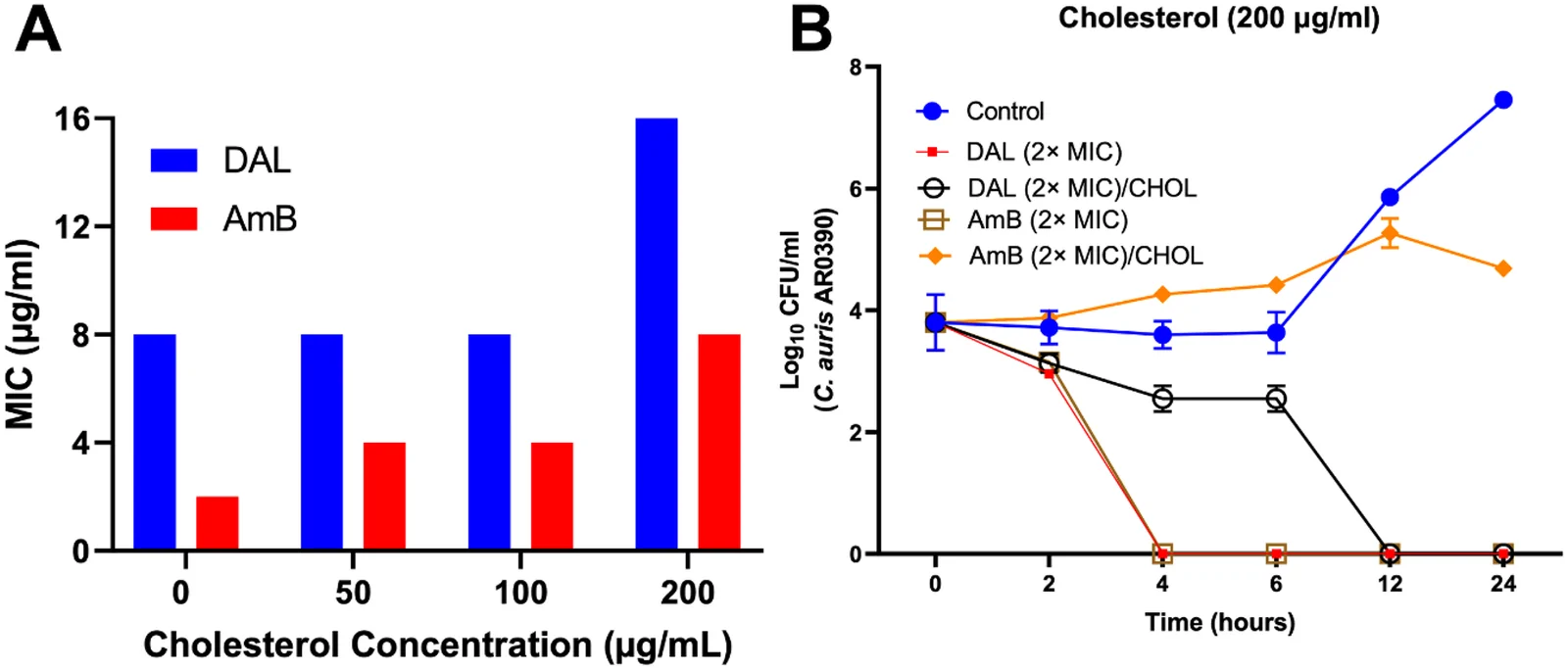
Effect of exogenous cholesterol (50-200 µg/mL) on the MICs (A) and time-kill (*n*  =  3) (B) of darapladib (DAL) and amphotericin B (AmB) at 2 × MIC against *C. auris* AR0390.

### *In vivo* efficacy of DAL in *C. elegans* infected with *C. auris*

We next evaluated the *in vivo* antifungal activity of DAL using a *C. elegans–C. auris* infection model. Infected worms were treated with DAL, ITC at 2 × MIC, or vehicle control, and fungal burden was quantified by CFU enumeration. DAL treatment reduced viable *C. auris* CFUs within infected worms to undetectable levels (below Log_10_ 1 CFU/mL) (Fig 8). In contrast, ITC produced a modest reduction of 1.87 log_10_ CFU compared to untreated controls, indicating substantially lower *in vivo* efficacy under the same conditions.

**Fig 8:**
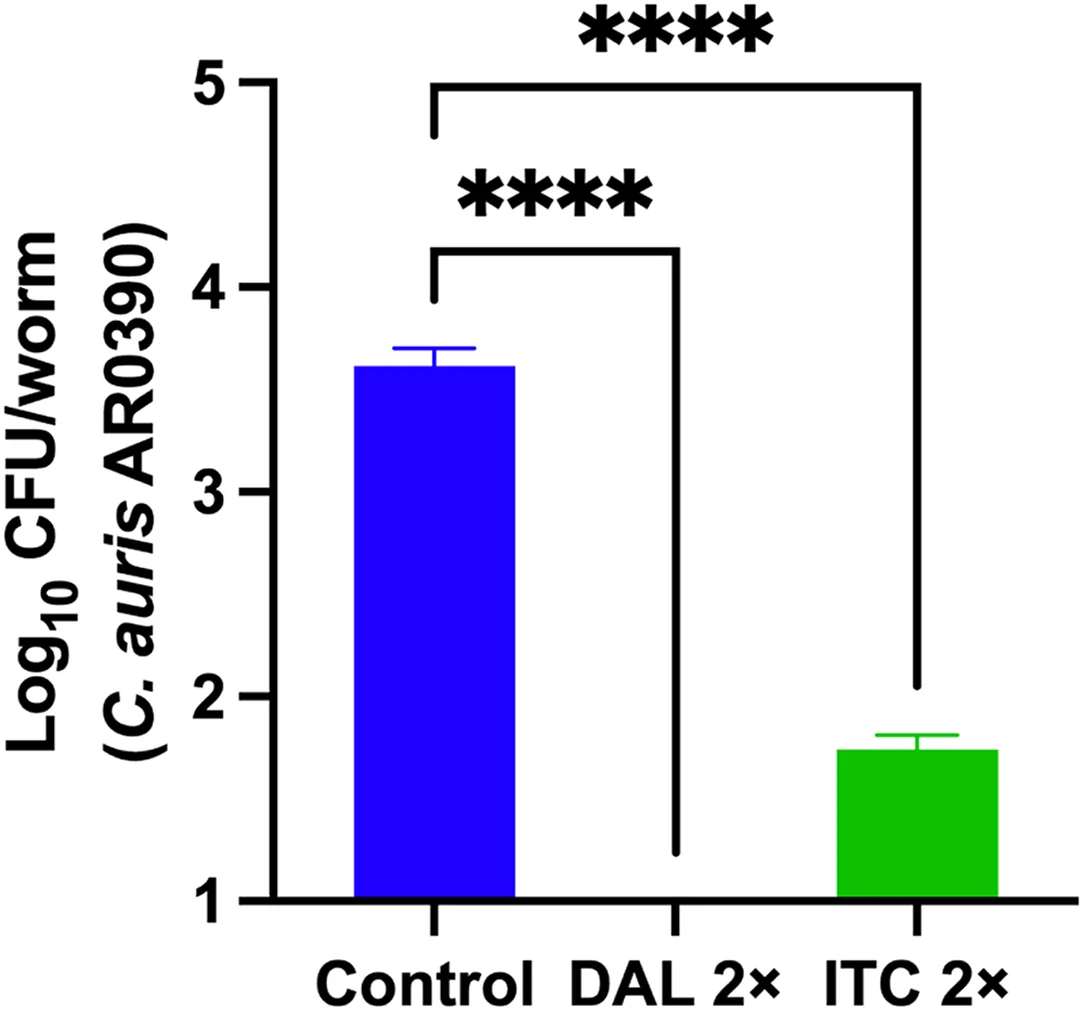
*In vivo* efficacy of darapladib (DAL), and itraconazole (ITC) in a *Caenorhabditis elegans* infection model of *C. auris.* L4-stage worms (average *n* = 100 worms per replicate) were infected with *C. auris* AR0390 for 4 hours, washed with PBS, and treated with DAL or ITC at 2 × their respective MIC. After 24 hours of treatment, the worms were homogenized, and the homogenates (*n*  =  3/treatment) were plated on YPD agar plates to quantify colony-forming units (CFU) per worm. The limit of detection was 1 log_10_ CFU/mL. Data are presented as means ± SD. Statistical significance was determined using a One-way ANOVA. **** = *P* value ≤ 0.0001.

## Discussion

Antifungal resistance has emerged as a global healthcare crisis, with an increasing number of pathogenic fungal species demonstrating resistance to existing antifungal treatments [2]. This problem is particularly acute for multidrug-resistant *C. auris*, which is associated with high mortality and limited treatment options. The urgent need for new antifungal agents is further compounded by the narrow therapeutic arsenal and toxicity of current antifungal drugs. In this study, we screened a library of 2,600 drugs and clinical compounds and identified DAL, a PLA2 inhibitor, as potent antifungal agent against multidrug-resistant *C. auris.* Interestingly, DAL exhibits fungicidal activity with rapid killing kinetics like polyenes. In addition, DAL exhibited synergistic activity with AmB, enhancing antifungal efficacy against the tested isolates of major clinically relevant fungal pathogens, including *Cryptococcus, Aspergillus* and mucormycosis*-*associated species, supporting the broad-spectrum activity of DAL in antifungal therapy. However, additional clinical isolates are needed to confirm its activity against these fungal species.

The rapid fungicidal activity of DAL prompted us to investigate its mechanism of action. While a previous study suggested that PLA2 inhibitors can interact with lipid bilayers [59], fungal membranes differ from mammalian membranes in that they contain ergosterol rather than cholesterol. Given this distinction, we hypothesized that DAL may preferentially interact with ergosterol in fungal membranes, resulting in nucleic acid leakage and cell death. Supporting this, supplementation with exogenous ergosterol resulted in a dose-dependent attenuation of DAL antifungal activity, like the effect observed with AmB. Time-kill assays further demonstrated that ergosterol partially rescued fungal growth and shifted DAL activity from fungicidal to fungistatic over 24 hours. These findings support an association between DAL activity and ergosterol but do not demonstrate direct physical binding between them. Thus, direct biochemical or biophysical binding studies will be required to determine whether this interaction occurs.

Importantly, our findings showed that cholesterol supplementation had a lesser effect on DAL activity than AmB, indicating that DAL has a weaker association with cholesterol. DAL also retains its fungicidal activity against *C. auris* in the presence of cholesterol, while AmB did not under the same conditions. Additionally, the combination DAL/AmB exhibited synergistic interaction at lower AmB concentrations, with Vero cell viability comparable to that of AmB alone. These findings suggest that DAL may reduce the AmB concentration required for antifungal efficacy. However, direct cholesterol binding and comprehensive mammalian toxicity studies are needed to determine DAL’s safety profile.

Reduced susceptibility of heterozygous *C. albicans* haploinsufficiency mutants (Log_10_  ≥  2) with disruptions in genes involved in the ergosterol biosynthesis pathways indicates that alterations in membrane sterol composition affect DAL antifungal activity. Thus, disruption of ergosterol biosynthesis may reduce the interaction between DAL and fungal membrane, thereby attenuating DAL antifungal activity compared to the parental strain. These findings are consistent with prior studies demonstrating that deletion or disruption of ergosterol biosynthesis genes alters membrane sterol content and reduces susceptibility to AmB, further supporting the critical role of ergosterol in mediating antifungal interactions with the fungal membrane [60–63].

We have confirmed the ability of DAL to cause membrane permeability and nucleic acid leakage of *C. auris* cells. Some antifungal agents are known to exert their antifungal mechanism of action through production of ROS [44], so we tested the ability of DAL to increase ROS levels in *C. auris*. Our findings showed that DAL (at 2 × MIC) elevated the ROS levels, suggesting the potential role of ROS in the antifungal activity of DAL. The synergistic interaction of DAL and AmB may result from DAL-induced membrane perturbation, potentially enhancing the membrane disrupting activity of AmB, leading to increased fungal killing. However, further studies are needed to establish the molecular basis of this interaction.

No reduction in *C. auris* susceptibility to DAL was detected after 16 serial passages. Although these findings are encouraging, additional studies involving several clinical isolates and extended serial passages are needed to further evaluate the potential for resistance development. Finally, we evaluated the *in vivo* efficacy of DAL in a *C. elegans* model, a widely used tool in antifungal drug development to evaluate the *in vivo* efficacy of potential therapeutics and assess the ability of antifungal compounds to reduce fungal burden [64,65]. DAL exhibited a great potency, reducing viable *C. auris* cells to below the limit of detection in the model. Further studies are needed to evaluate DAL pharmacokinetics, mammalian toxicity, and therapeutic efficacy, both alone and in combination with AmB.

In summary, our results suggest that DAL is a promising antifungal compound as it exhibits fungicidal, broad-spectrum, and *in vivo* efficacy against the tested fungal isolates. No development of resistance to DAL has been observed against *C. auris*. The chemical structure of DAL, as a phospholipase inhibitor, provides a foundation for potential modifications to enhance its efficacy, prevent resistance emergence, and improve pharmacokinetic properties. Further studies to determine the feasibility of PLA2 inhibitors as antifungal therapeutic agents are warranted.

## Supporting information

S1 Table Sources, and clades of *Candida auris* isolates used in this study. (DOCX)

S2 Table Sources of other fungal isolates used in this study. (DOCX)
