# Supplementary material for: Repurposing the phospholipase A2 inhibitor, darapladib, to combat multidrug-resistant fungal pathogens, including Candida auris
Source: PLoS One. 2026 Oct 26;21(10):e0361366. doi: 10.1371/journal.pone.0361366 (PMC13704847; doi:10.1371/journal.pone.0361366)
Supplement: Supplementary file 1 [file pone.0361366.s001.docx]

**Table S1**. Sources and clades of *Candida auris* isolates used in this study.

| **Fungal isolate** | **Clade** | **Source** |
| --- | --- | --- |
| ***C. auris* AR0381** | II | Ear |
| ***C. auris* AR0382** | I | Wound |
| ***C. auris* AR0383** | III | Blood |
| ***C. auris* AR0384** | III | Blood |
| ***C. auris* AR0385** | IV | Blood |
| ***C. auris* AR0386** | IV | Blood |
| ***C. auris* AR0387** | I | Blood |
| ***C. auris* AR0388** | I | Blood |
| ***C. auris* AR0389** | I | Bronchoalveolar lavage (BAL) Fluid |
| ***C. auris* AR0390** | I | Pus |
