# Supplementary material for: Repurposing the phospholipase A2 inhibitor, darapladib, to combat multidrug-resistant fungal pathogens, including Candida auris
Source: PLoS One. 2026 Oct 26;21(10):e0361366. doi: 10.1371/journal.pone.0361366 (PMC13704847; doi:10.1371/journal.pone.0361366)
Supplement: Supplementary file 2 [file pone.0361366.s002.docx]

**Table S2**. Sources of other fungal isolates used in this study.

| **Fungal isolate** |  |
| --- | --- |
|  | **Source** |
| ***C. albicans* ATCC® 64124** | Human mouth swab |
| ***C. krusei* ATCC®34135** | Human clinical isolate, Minnesota |
| ***C. parapsilosis* ATCC® 22019** | Human clinical isolate, Puerto Rico |
| ***C. tropicalis* ATCC® 1369** | Not applicable |
| ***C. neoformans* NR-41298** | Human cerebrospinal fluid, China, February 2012. |
| ***C. neoformans* NR-41300** | Human cerebrospinal fluid, China, May 2011. |
| ***C. gattii* NR-43208** | Human during the Vancouver outbreak, Canada |
| ***Rhizopus arrhizus* var. delemar CBS 120593** | Human lung, France. |
| ***Mucor indicus* CBS123974** | Human gastrointestinal infection, Germany. |
